# Supplementary material for: Prevalence of scabies and associated factors among school age children in Qoloji IDP in Babile district, Somali, Eastern Ethiopia
Source: PLoS One. 2025 Apr 1;20(4):e0320675. doi: 10.1371/journal.pone.0320675 (PMC11960965; doi:10.1371/journal.pone.0320675)
Supplement: Attachment — Submitted filename: COVER LETTER to Editors of PLOS ONE.docx [file pone.0320675.s003.docx]

Dear Editors of the **Journal of PLOS ONE**

I am writing to you as a reviewer for the manuscript titled “**PREVALENCE OF SCABIES AND ASSOCIATED FACTORS AMONG SCHOOL AGE CHILDREN IN QOLOJI IDP IN BABILE DISTRICT, SOMALI, EASTERN ETHIOPIA**." I have carefully reviewed the submission and I would like to provide some feedback to the authors to enhance the clarity and quality of their research article

The study addresses an important topic related to “PREVALENCE OF SCABIES AND ASSOCIATED RISK FACTORS AMONG SCHOOL AGE CHILDREN. While the research findings are valuable for this journal, some improvements were found to be important to better express the study. I have provided detailed feedback to the authors on various sections of the manuscript, including the abstract, methods, results, introduction, and conclusion. By addressing these points, the authors can strengthen their research article and contribute more effectively to the field of public health.

I am happy to review this submission and contribute to the peer-review process at the **Journal of PLOS ONE**. I look forward to seeing the revised manuscript and commend the authors for their dedication to advancing knowledge in this field.

Thank you for considering my feedback and for your commitment to publishing high-quality research in **Journal of PLOS ONE**

Best regards,

[Abdulaziz Abrahim], Reviewer at the **Journal of PLOS ONE**
