# Supplementary material for: Prevalence of scabies and associated factors among school age children in Qoloji IDP in Babile district, Somali, Eastern Ethiopia
Source: PLoS One. 2025 Apr 1;20(4):e0320675. doi: 10.1371/journal.pone.0320675 (PMC11960965; doi:10.1371/journal.pone.0320675)
Supplement: Attachment — Submitted filename: Journal of PLOS ONE.docx [file pone.0320675.s002.docx]

**Journal of PLOS ONE**

**Title: PREVALENCE OF SCABIES AND ASSOCIATED FACTORS AMONG SCHOOL AGE CHILDREN IN QOLOJI IDP IN BABILE DISTRICT, SOMALI, EASTERN ETHIOPIA.**

**Manuscript Numbe**r: **PONE-D-24-34114**

**Article Type**: **Research Article**

**Dear authors,**

Thank you for submitting your research article to the Journal of PLOS ONE. I appreciate the effort you have put into conducting this study and sharing this interesting findings with the scientific community. To improve the quality of your research I put some comments and suggestion below.

**Reviewer Comments**

**Abstracts**

**Comment 1:** In Ethiopia now a day there are social unrest, conflict and human made disasters for the sake of this there are displacement of the population from one place to another to relieve this problem and it is the commonest skin diseases of the population lives in internal displaced people. I think it’s well if the authors changes above statements into “In Ethiopia now day there are social unrest, conflict and human made disasters to relieve this problem there are displacement of the population from one place to another”. Also delete the statements “it is the commonest skin diseases of the population lives in internal displaced people and why the authors relate the scabies with conflicts or social unrest, rather its better if the authors relate scabies with hygienic practice, living standards etc. If the research’s conducted in displacement camps, it’s ok.

**Comment 1:** Dear researchers I could not seen the significance of this study/why this study was needed?. Briefly mention your significance. Also merge your study objective with your background.

**Comment 2:** The data was collected by using observation (clinical investigation), structured questionnaires.

**Comment 3:** Data was coded, entered and cleaned using with epi data version 3.1, and exported and analysis was done by using SPSS statistical software package version 22. Rearrange above statements, and shorten “Logistic regression analysis was used to identify factors associated with scabies. Findings were presented by using 95% CI of Crude Odds Ratios (COR) and Adjusted Odds Ratios (AOR). To declare statistical significance, a p-value of less than 0.05 was used”

**Comment 4:** in conclusion “Health education on personal hygiene (frequent bathing, washing clothes, and changing clothes regularly), avoiding sharing clothes with others, avoiding contact, sleeping with scabies-ill people, and sharing beds with others”. Simply this list of risk factors, why this factors listed here without any messages. I recommended that the authors mentioned why He list them i.e. his recommendation or the practice of community. Keywords must written in alphabet orders.

**INTRODUCTION**

Dear authors it’s interesting if you add the prevalence of scabies in Ethiopia and the study area and also add information about taxonomy of mites and also mention the group organism in which scabies belongs. Dear authors I have seen in your abstracts the scabies is a disease that do not discriminate age, sex, social class etc., but in your introduction there is contrasting idea make clear why this is possible in the introduction.

In this part many sentence is reported without referenced, add necessary references.

Why the authors write some parts of introduction using different text and fonts.

**Comment 5:** Line number 50-51 “Mite is the common cause of this communicable infectious disorder of the skin” change into “Mite is the common **causative agents** of this communicable infectious disorder of the skin

**Comment 6:** Line number 51-51: And the mites are not noticeable with the naked eye but can be seen with microscope” delete or add to diagnosis information.

**Comment 7:** Line number 56-57, the onset of the symptoms depends on the level of infection; a scabies patient normally has 12 mites present at any given time. What is 12, is the numbers of mites per infected site/wound or the number of mites on/in the body of infected person? Clarify.

**Comment 8:** Include Line number 99-111, under the first paragraph and also there is repetition of sentence in your introduction, For example line number 82-85 with line number 112-`13.

**Material and Methods**

Dear authors what is the difference b/n the target population and study population, I think its good if the authors merge them.

**Comment 9:** Line number 157: a large majority of displaced persons change the word persons into people and also put the study period at the starts of this parts. Line 171 change the word conducted into employed.

**Result**

A total of 413 participants were included in the study, making a response rate of 100%. Is your sample size 413 or 422? Make sure. I have seen throughout your result parts the percentage of the response if written before the frequency, also in some area you have written only percentage where as in others both frequency and percentages were reported. Dear authors I recommended you its better if you rewrite the results of your study in similar manners (example 336 (79.6%) or (79.6%), but not possible to write 79.6% (336), although this may not changes what intended to be mentioned, it may reduce the attractiveness and clarity of your paper.

**Comment 10:** Line 303, more than three-quarters of the sample (79.6%), had a family size greater than or equal to five, rather to say sample, changes to **participants, and correct “t**he majority of 396 (93.8%) of the respondents use wells or springs as a source of water for personal hygiene in the study area” as **“t**he majority of respondents (396 (93.8%) use wells or springs as a source of water for personal hygiene in the study area. Line number 306-307: “Regarding the water source, nearly almost all (93.8%) of households use improved water sources”. Clarify the improved water (i.e. in quality or improved in quantity).

**Comment 11:** Line number 307-308: 7.2% (31) of the study participants have a history of contact with previous scabies patients in the household. Use only percentages, if you include frequency include in all results, but before the percentages outside of the brackets.

**DISCUSSION**

I appreciated your discussion and for your time to prepare such well organized and informative discussion. But to make your discussion more interesting I have put some recommendation and correction below.

**Comment 12:** Line number 316: 14.92 is frequency or percentages/mean and dear authors why you do not use similar significant figures (example 12.93% other hands use 16.4%)

**Comment 13:** The authors mentioned that your study is comparable with study conducted in Cameroon 17.8% (18), Nigeria 13.3% (19), Arbaminch district, south part of Ethiopia 16.4 (12), Raya Alamata, which is 12.93% (5). Based on what, like your results you have to mention what they reported, here you are list only percentages. Correct as “comparable with study conducted in Cameroon 17.8% (18), Nigeria 13.3% (19), Arbaminch district, south part of Ethiopia 16.4% (12), Raya Alamata, Ethiopia which is 12.93% prevalence (5)

**Conclusion and Recommendation**

Prepared well.

Generally the document needed some grammar and language improvement

Thank you for considering these suggestions to enhance the quality and clarity of your research article. I look forward to seeing the revised manuscript.

**Best regards,**

**[Abdulaziz Abrahim]**

**Reviewer for the Journal of PLOS ONE**
