# Supplementary material for: Prevalence of scabies and associated factors among school age children in Qoloji IDP in Babile district, Somali, Eastern Ethiopia
Source: PLoS One. 2025 Apr 1;20(4):e0320675. doi: 10.1371/journal.pone.0320675 (PMC11960965; doi:10.1371/journal.pone.0320675)
Supplement: Attachment — Submitted filename: Response to Reviewers.docx [file pone.0320675.s007.docx]

**Reviewer Comments and Author Responses**

**Dear Editor and Reviewer,**

Thank you for your comments. We have made all the necessary corrections required.

**Reviewer #2:**

**Reviewer Comments:** You tried addressing the previous comment; however, there is still a citation problem, e.g., line 301, title 4.4.1 formula, etc.

**Author Response:** We have corrected this based on your comment. The citations are now grammatically correct.
